# Comprehensive assessment of the management of acute cholecystitis in Scotland: population-wide cohort study

**DOI:** 10.1093/bjsopen/zrad073

**Published:** 2023-08-14

**Authors:** Mohamed Bekheit, Sendhil Rajan, Jared M Wohlgemut, Angus J M Watson, George Ramsay

**Affiliations:** Department of General Surgery, NHS Grampian, Aberdeen, UK; HPB Surgery Unit, Integrated Centres of Excellence, Elite Healthcare, Alexandria, Egypt; Department of General Surgery, NHS Grampian, Aberdeen, UK; Centre for Trauma Sciences, Blizard Institute, Queen Mary University of London, London, UK; Department of Surgery, Raigmore Hospital, Inverness, UK; Department of General Surgery, NHS Grampian, Aberdeen, UK; Health Services Research Unit, University of Aberdeen, Aberdeen, UK

## Abstract

**Background:**

Acute cholecystitis is one of the most common diagnoses presenting to emergency general surgery and is managed either operatively or conservatively. However, operative rates vary widely across the world. This real-world population analysis aimed to describe the current clinical management and outcomes of patients with acute cholecystitis across Scotland, UK.

**Methods:**

This was a national cohort study using data obtained from Information Services Division, Scotland. All adult patients with the admission diagnostic code for acute cholecystitis were included. Data were used to identify all patients admitted to Scottish hospitals between 1997 and 2019 and outcomes tracked for inpatients or after discharge through the unique patient identifier. This was linked to death data, including date of death.

**Results:**

A total of 47 558 patients were diagnosed with 58 824 episodes of acute cholecystitis (with 27.2 per cent of patients experiencing more than one episode) in 46 Scottish hospitals. Median age was 58 years (interquartile range (i.q.r.) 43–71), 64.4 per cent were female, and most (76.1 per cent) had no comorbidities. A total of 28 741 (60.4 per cent) patients had an operative intervention during the index admission. Patients who had an operation during their index admission had a lower risk of 90-day mortality compared with non-operative management (OR 0.62, 95% c.i. 0.55–0.70).

**Conclusion:**

In this study, 60 per cent of patients had an index cholecystectomy. Patients who underwent surgery had a better survival rate compared with those managed conservatively, further advocating for an operative approach in this cohort.

## Teaser

We describe, at a population level, the management of cholecystitis in Emergency General Surgery in Scotland, UK. Herein, we observe that only 60 per cent of individuals will have an emergency cholecystectomy on index admission. We also describe the risk factors associated with 90-day mortality after admission.

## Introduction

Acute cholecystitis (AC, gallbladder inflammation) is one of the most common causes of emergency general surgery (EGS) admission across the world. This diagnosis accounts for between 3 and 10 per cent of all patients presenting with abdominal pain^1^. The management of cholecystitis can either be operative (by means of a cholecystectomy) or by a conservative approach (using analgesia and antibiotics, with or without a cholecystostomy drain). The decision to undertake one treatment plan over another is multifactorial with surgeon, facility, and patient factors influencing the choice. At present, which approach is utilized most in different healthcare systems has not been fully described.

There are several consensus guidelines that recommend early cholecystectomy as the first-line management of this condition (if co-morbidities allow)^2,3^. However, the evidence for this advice has been developed from RCTs and has not considered the real-world challenges of complexities of surgical skillsets and competing EGS cases. In England, fewer than 50 per cent of patients will receive an operation during the index admission^4,5^. Whether this reflects practice in other countries remains unclear. Despite the previous recommendations, the non-operative management of AC has been reported to be safe with less than a quarter of patients re-presenting with symptoms^6^. Clinicians and patients need to make decisions around two management options, both of which are perceived to be acceptable treatments for this condition.

The management of patients with AC needs to be taken in the context of an EGS system, which is invariably busy with multiple patients with a range of acute pathologies, requiring acute care concordantly. As a condition in which conservative management is feasible, and with limited theatre capacity in some settings, it may be that external pressures associated with the management of patients with other EGS conditions necessitate a non-operative approach for individuals with AC. The competence and confidence of a surgical team to undertake a cholecystectomy in the acute setting also needs to be factored in to management decisions.

Previous studies, exploring outcomes after cholecystectomy in Scotland, have predominantly assessed operative outcomes^7^. In the context of a condition where a non-operative approach is often utilized, any description of the real-world management of AC must include those individuals managed conservatively. Such work would need to compare the survival during their index admission with cholecystitis, but also after EGS discharge. Evaluation of the whole cohort, including those managed without an operation, is not well studied.

The primary aim of the study was to describe the EGS management of AC in Scotland. A secondary aim was to determine the 90-day mortality rates of patients with acute cholecystitis, comparing those who had an operation upon index admission with those who were managed conservatively.

## Methods

### Study design

This was a population cohort analysis.

### Data source

The Scottish National Health Service (NHS Scotland) records data on all hospital admissions through its Information Services Division (ISD). Each patient is assigned a unique number (Community Health Index (CHI) number) at their first contact with NHS Scotland services. The ISD data can also be linked to death records, and hence permits mortality analysis even after patients have been discharged from the acute setting, or if they are re-admitted in another facility in the country.

Linked data was provided covering the hospital episodes, hospital type, distance from domicile or admission source to the hospital of admission, 8-fold Scottish Urban Rural Classification (SURC), and Scottish Index of Multiple Deprivation (SIMD) quintile. In addition, data were collected on patient gender, age at each admission, 5- and 10-year Charlson co-morbidity index (CCI), date of admission and discharge, as well as the treatments provided.

### Participants

The study included a cohort of patients aged over 16 years with information on their index hospital admissions with acute cholecystitis between 1997 and 2019. Patients were followed up for 90 days after discharge. Patient admissions were identified based on the International Statistical Classification of Diseases and related health problems, 10th revision (ICD-10)^8^ diagnostic code for acute cholecystitis (K80.x, K81.x, K82.x). Patients were excluded if they had more than a single diagnosis code, including pancreatitis, cholangitis, choledocholithiasis, jaundice, or other concomitant pathology irrelevant to cholelithiasis.

### Operational definitions

This study included cholecystectomy performed during the index admission, which is defined as the first acute presentation with acute cholecystitis, regardless of the duration of the associated hospital stay. The rurality index was calculated using the 8-fold SURC and distance to nearest referral centre type was utilized as a covariate^9^. Urban–rural residency was demonstrated as an influential factor on surgical decision-making, and therefore, included as a covariate^10^. The SIMD was used as a surrogate for the patient’s socioeconomic circumstances^11,12^. The exposure of interest was the outcome of the admission in terms of a conservative *versus* interventional approach during the index admission. The primary outcome measure was 90-day mortality^13,14^.

### Covariates

Since the outcome is influenced by composite dimensions, the study used a hierarchical logistic regression model to evaluate the influence of the patient, hospital level variables on 90-day mortality in relation to the index admission.

The distribution of the covariates of patient characteristics based on them receiving an operation against their individual mortality risk was assessed. Potential confounders at the patient (such as age, sex, CCI, SURC, SIMD) and hospital (such as the proportion of patients who had an operation for acute cholecystitis during the index admission at each hospital, and whether the hospital was a teaching/non-teaching one) levels were identified for inclusion in hierarchical logistic regression models to account for covariates at patient and hospital levels.

### Data handling and risk adjustment

The proportion of patients with missing data was low (less than 5 per cent), so imputation was not used. Risk-adjusted outcomes were expressed as odds ratios (OR) with 95 per cent c.i. We evaluated the distribution of the covariates of patient characteristics based on receiving an operation against their individual mortality risk. A standard difference of less than 0.1 was used to support covariate balance.

### Statistical analysis

Analyses were performed using SPSS Version 25. Medians and interquartile ranges (i.q.r.) were calculated for continuous variables, frequencies were used to summarize discrete variables. Proportions were compared using the chi-squared test; medians were compared using nonparametric tests. A threshold of *P* <0.05 was considered statistically significant. Variables included in the model were tested for collinearity before their final inclusion in the models.

### Study conduct

This study was approved via the Public Benefit and Privacy Panel for Health and Social Care (PBPP 2021-0037), and did not require further research ethics approval. The STROBE guidelines informed manuscript preparation (*Table S1*)^15^.

## Results

### Cohort description

A total of 47 558 patients with acute cholecystitis were admitted to 46 EGS units across Scotland between January 1997 and April 2019. These patients accounted for 58 824 episodes, with 27.2 per cent of patients encountering more than one episode (ranging from 1 to 11 episodes). The median age of the study population was 58 years (i.q.r. 43–71), and 64.4 per cent were female. The majority of patients (76.1 per cent) did not have any co-morbidity (median CCI with a 5-year and 10-year lookback was 0 (ranging from 0 to 18)). Stratified by SIMD quintiles, the most deprived areas were the source of 25.8 per cent of the total hospital episodes, while the least deprived areas were the source for the lowest number of hospital episodes at 14.1 per cent. Regarding rurality, settlements with 10 000–124 999 inhabitants (SURC = 2) were the largest source with 37.8 per cent of all hospital episodes, followed by the urban larger areas (SURC = 1; >125 000 inhabitants) representing 32.8 per cent of the total recorded hospital episodes. *Table 1* lists the patient demographics in this cohort.

**Table 1:** Description of the cohort

Baseline characteristic	*n*
No. of patients	47 558
No. of episodes	58 824
No. of patients admitted only once	34 615 (72.8%)
Median age (i.q.r.)	58 years (i.q.r. 43–71)
**Sex**	
Male	16 931 (35.6%)
Female	30 627 (64.4%)
Patients who had an operation during emergency care	33 415 (70.3%)
Operation at index admission	28 714 (60.4%)
Operation at subsequent admission	4674 (9.8%)
Overall duration of hospital stay in number of days (i.q.r.)	4 (2–6)
Missing data	42 (0.08%)

### Operative *versus* non-operative approach

In this cohort, 28 741 (60.4 per cent) patients had an operative intervention during the index admission, and 4 674 (9.8 per cent) had an operation during a subsequent emergency admission (*Table 2*). The median interval between admission to operation was 1 day (i.q.r. 0–3 days).

**Table 2:** Operative *versus* conservative management

	Operative management	Conservative management
No. of patients	28 714 (60.4%) at index admission	18 844 (39.6%)
Median age (i.q.r.)	55 (27–83)	60 (33–87)
Female	19 034 (66.2%)	9860 (52.3%)
CCI (i.q.r.)	0 (0–1)	0 (0–1)
Median interval between admission to operation	1 day (i.q.r. 0–3)	Not applicable
Median duration of hospital stay (index admission)	4 days (i.q.r. 3–7)	3 days (i.q.r. 1–5)
Mortality (at 90 days from admission)	459 (1.6%)	452 (2.4%)

Baseline characteristics, in-hospital stay and 90-day survival of patients having had a cholecystectomy operation and those who have been managed conservatively. CCI, Charlson Comorbidity Index; i.q.r., interquartile range.

Patients who underwent cholecystectomy during their index admission were younger (median 55 years, i.q.r. 27–83) compared with those who were managed non-operatively (median 60 years, i.q.r. 33–87). Women were more likely to have an operation during their index admission (OR 1.23, 95 per cent c.i. 1.18–1.27). Patients with more co-morbidities (by CCI lookback) were similarly less likely to have an operation during index admission (OR 0.84, 95 per cent c.i. 0.83–0.85). However, a CCI of median 0 (i.q.r. 0–1) was recorded for both operated and conservatively managed patients at both 5- and 10-year lookback. Patients living farthest from the hospital were also slightly more likely to get an operation during their index admission (OR 1.002, 95 per cent c.i. 1.001 to 1.002).

### Duration of hospital stay

The median overall and postoperative hospital stay is summarized in *Table 2*. Patients who were not operated on during an emergency admission tended to have a shorter duration of hospital stay (median 3 days, i.q.r. 1–5 days), than those who had a cholecystectomy during their index admission (median 4 days, i.q.r. 3–7 days). Patients who had an operation remained longer in hospital (OR 6.1, 95 per cent c.i. 5.6–6.8).

### Mortality

#### Association of 90-day mortality with patient characteristics

**Fig. 1:**
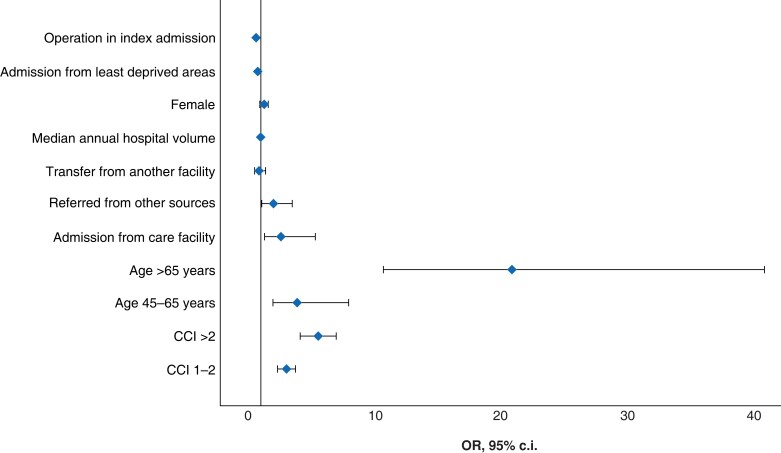
**Forest plot of factors influencing mortality for all admissions. Presenting odds ratios (OR) and 95 per cent c.i.** .

Adjusted for patient characteristics, women had higher odds of 90-day mortality compared with males (OR 1.28, 95 per cent c.i. 1.06–1.55). Mortality increased with age, as patients aged between 45 and 65 years, and more than 65 years, had increased odds of mortality (OR 3.9, 95 per cent c.i. 1.95–7.95; OR 20.9, 95 per cent c.i. 10.7–40.96 respectively), compared with patients less than 45 years of age. Compared with patients with no co-morbidities (CCI = 0), patients with co-morbidities had higher odds of mortality (CCI = 1–2: OR 2.96, 95 per cent c.i. 2.35–3.73; CCI >2: OR 5.4, 95 per cent c.i. 4.2–6.97). Compared with patients admitted from a domicile environment (that is home), patients who were admitted from a care facility had higher odds of mortality (OR 2.6, 95 per cent c.i. 1.3–5.3), while patients transferred from another medical facility had no significant mortality difference (OR 0.86, 95 per cent c.i. 0.53–1.39). Admission from the least deprived areas had lower odds of mortality compared with those admitted from the most deprived areas (OR 0.74, 95 per cent c.i. 0.59–0.93). Rurality did not significantly affect mortality. Odds ratios are summarized in *Table 3* and *Fig. 1*.

**Table 3:** Factors influencing 90-day mortality for all admissions (hierarchical logistic regression model)

Attribute	OR	Lower 95% c.i.	Upper 95% c.i.
**Sex**			
Male	Ref		
Female	1.28	1.06	1.55*
**Age**			
Age <44	Ref		
Age 45–65 years	3.9	1.95	7.95*
Age >65 years	20.9	10.7	40.96*
**CCI**			
CCI–0	Ref		
CCI 1–2	2.96	2.35	3.73*
CCI >2	5.4	4.2	6.97*
**Admission**			
Admission from home	Ref		
Admission from care facility	2.6	1.3	5.3*
Transfer from another facility	0.86	0.53	1.39
Referred from other inpatient wards	1.98	1.1	3.5*
**Admission and deprivation**			
Admission from most deprived areas	Ref		
Admission from least deprived areas	0.74	0.59	0.93*
**Operation on index admission**			
No operation on index admission	Ref		
Operation on index admission	0.62	0.55	0.7*
Median annual hospital volume	0.99	0.998	0.999*

*Denotes *P* <0.001. CCI, Charlson Comorbidity Index; i.q.r., interquartile range.

#### Association between mortality and operation

After adjusting for patient and hospital characteristics, having an operation during the index admission had a lower risk of mortality compared with non-operative management (OR 0.62, 95 per cent c.i. 0.55–0.7). The 90-day mortality at index admission in the non-operated patients was 2.5 per cent compared with 1.6 per cent among those that had a cholecystectomy (*P* <0.001). A total of 0.3 per cent of all patients had a recorded in-hospital mortality (95 per cent c.i. 0.25–0.34). Inpatient mortality varied significantly between hospital types (*P* = 0.002). However, there was no significant difference in 90-day mortality from operation (OR 0.88, 95 per cent c.i. 0.72–1.08) or 90-day mortality from admission (OR 0.95, 95 per cent c.i. 0.83–1.08) between teaching hospitals and large general hospitals.

## Discussion

Surgical literature tends to concentrate on outcomes for patients that have had operations. Patients who have diagnoses with potentially curative surgical treatments, who are managed conservatively, are less studied^16^. This is particularly relevant in EGS care where only approximately 25 per cent of patients admitted to hospital will have a procedure^17^. The majority of patients, therefore, are managed conservatively. However, the outcomes for specific diagnoses, such as AC, have not been fully explored, particularly when there are operative and non-operative options for treatment.

AC is a condition where the approach can be surgical or conservative. The decision to adopt one approach over the other varies between clinicians^18^. This study presents the management of 47 558 patients with AC in Scotland over the past two decades. It assesses the relationship between the therapeutic strategy and 90-day patient mortality, which is one of the largest cohort studies available in the literature to date. In this cohort, 60 per cent of patients had an operation on their index admission and, when controlled for other patient factors, this approach had a reduced 90-day mortality rate. In summary, a reduced mortality in patients who had an operation was observed; acute cholecystitis appears to have been optimally treated by an operation in the EGS setting, in keeping with the current guidelines.

Other characteristics that were associated with mortality included advancing age, co-morbidities, admission from a care facility, and being a woman. Each of these characteristics have been identified to contribute to higher complication rates in previous cohort series concentrating on surgical approaches to treat cholecystitis^19,20^. This study unsurprisingly demonstrates that these factors are also significant in determining outcome in those who had conservative management.

There was lower mortality in those who underwent cholecystectomy on index admission compared with those treated non-operatively, after risk adjustment. This indicates that operative intervention is beneficial in reducing the mortality risk in accordance with other studies^21^. The only exceptions are patients who are ASA IV or above^22^. The results are consistent with the findings from a previous series in other settings^5^. Often, increasing age and co-morbidity are used to avoid operations in surgical care. However, in the context of cholecystitis, the CHOCOLATE (laparoscopic cholecystectomy versus percutaneous catheter drainage for acute cholecystitis in high risk patients) study demonstrated that even in this context, a cholecystectomy is superior to a percutaneous drain^23^. On an individual patient-level basis, cholecystitis is best managed by removal of the gallbladder.

Cholecystectomy during the index admission is a preferred approach in current guidelines for most patients^2,3^. However, despite this guidance being available for some time, it is striking that only 60 per cent of patients have this approach adopted. This observation is important for resource management of EGS stakeholders. It is likely that the reasons for such a low operative rate are multiple, with surgeon expertise, availability of resources, and competing caseloads requiring operative intervention being key influencing factors. The operative rate is higher than a study of patients in England (2021), where 48.9 per cent of a total 99 139 patients underwent a cholecystectomy within 1 year of index admission^5^.

A total of 1.93 per cent of those individuals in this cohort died within 90 days of their diagnosis and EGS admission. This is higher than previously perceived and highlights the severity of this common condition in EGS care. It is also important to recognize this mortality rate and it is particularly relevant when discussing potential outcomes with patients and family members.

This study has some limitations. The NHS Scotland patient data set is one of the strongest national data sets in the world. Through the use of the CHI number, inpatient stay can be accurately linked to discharge diagnosis, operations undertaken, and subsequent survival (whether death occurred in hospital or in the community). However, the data set did not record the date of symptom onset, rates of cholecystostomy, or the preoperative severity assessment of cholecystitis, which have previously demonstrated correlation with outcomes^24^. Furthermore, postoperative morbidity, such as bile leak, bile duct injury, or hernia rate, was not analysed in this study. Future studies may look specifically at the relationship between hospital type and morbidity. Post-cholecystectomy morbidity has previously been shown to influence mortality^25^. Data are not available on subsequent elective cholecystectomy operation meaning that the outcomes were based on index cholecystectomy rates.

## Supplementary Material

zrad073_Supplementary_Data

## Data Availability

These data were provided to us through Information Services Division Scotland of NHS Scotland after approvals were obtained. They were analysed in the NHS Scotland Data Safehaven. As such, they are not available to be provided by the authors but are available through ISD Scotland.
