# Supplementary material for: Comprehensive assessment of the management of acute cholecystitis in Scotland: population-wide cohort study
Source: BJS Open. 2023 Aug 14;7(4):zrad073. doi: 10.1093/bjsopen/zrad073 (PMC10424165; doi:10.1093/bjsopen/zrad073)
Supplement: zrad073_Supplementary_Data [file zrad073_supplementary_data.docx]

**Title:**

**Comprehensive assessment of the management of acute cholecystitis in Scotland: A population wide cohort study of 47,558 patients**

**Authors:**

Mohamed Bekheit, PhD, FRCS^1,2^ [dr_mohamedbekheit@hotmaill.com](mailto:dr_mohamedbekheit@hotmaill.com)

Sendhil Rajan, MCh, MRCS, FEBS ^1^ [sendhil.rajan@nhs.scot](mailto:sendhil.rajan@nhs.scot)

Jared M Wohlgemut, MSc, MRCS ^3^ [jwohlgemut@nhs.net](mailto:jwohlgemut@nhs.net)

Angus JM Watson, MD, FRCS^4^ [angus.watson@nhs.scot](mailto:angus.watson@nhs.scot)

George Ramsay, PhD, FRCS^1,5^ [george.ramsay@abdn.ac.uk](mailto:Ggeorge.ramsay@abdn.ac.uk)

^1^ Department of General Surgery, NHS Grampian, Aberdeen, AB252ZN, UK

^2^ HPB Surgery Unit, Integrated centres of Excellence, Elite Healthcare, Alexandria, Egypt.

^3^ Centre for Trauma Sciences, Blizard Institute, Queen Mary University of London, 4 Newark Street, Whitechapel, London, E1 2AT, UK

^4^ Raigmore Hospital, Perth Road, Inverness, IV23UJ, UK

^5^ Health Services Research Unit, University of Aberdeen, AB252ZD, UK

*Keywords: Acute Cholecystitis, Cholecystectomy, Emergency General Surgery*

Corresponding author:

**Dr George Ramsay PhD FRCS**

Health Services Research Unit

University of Aberdeen

Health Sciences Building

Foresterhill

Aberdeen AB25 2ZD

Email: [george.ramsay@abdn.ac.uk](mailto:george.ramsay@abdn.ac.uk)

ORCID ID: <https://orcid.org/0000-0001-9862-2323>

Twitter: @GeoRamsay

**Supplementary Materials - Index**

| **Supplementary Figures and Tables** |  |
| --- | --- |
| STROBE Checklist | *page 18-19* |
| **References** | *page 13-14* |
|  |  |

**Supplementary Figures and Tables**

**Supplementary Table 1:** STROBE Checklist

STROBE Statement—Checklist of items that should be included in reports of ***cohort studies***

|  | Item No | Recommendation | Page |
| --- | --- | --- | --- |
| **Title and abstract** | 1 | (*a*) Indicate the study’s design with a commonly used term in the title or the abstract | 2 |
|  |  | (*b*) Provide in the abstract an informative and balanced summary of what was done and what was found | 2 |
| Introduction | | |  |
| Background/rationale | 2 | Explain the scientific background and rationale for the investigation being reported | 3-4 |
| Objectives | 3 | State specific objectives, including any prespecified hypotheses | 4 |
| Methods | | |  |
| Study design | 4 | Present key elements of study design early in the paper | 5-7 |
| Setting | 5 | Describe the setting, locations, and relevant dates, including periods of recruitment, exposure, follow-up, and data collection | 5-6 |
| Participants | 6 | (*a*) Give the eligibility criteria, and the sources and methods of selection of participants. Describe methods of follow-up | 5 |
|  |  | (*b*) For matched studies, give matching criteria and number of exposed and unexposed | NA |
| Variables | 7 | Clearly define all outcomes, exposures, predictors, potential confounders, and effect modifiers. Give diagnostic criteria, if applicable | 6 |
| Data sources/ measurement | 8* | For each variable of interest, give sources of data and details of methods of assessment (measurement). Describe comparability of assessment methods if there is more than one group | 5-7 |
| Bias | 9 | Describe any efforts to address potential sources of bias | 5-7 |
| Study size | 10 | Explain how the study size was arrived at | 5 |
| Quantitative variables | 11 | Explain how quantitative variables were handled in the analyses. If applicable, describe which groupings were chosen and why | 5-7 |
| Statistical methods | 12 | (*a*) Describe all statistical methods, including those used to control for confounding | 5-7 |
|  |  | (*b*) Describe any methods used to examine subgroups and interactions | 5-7 |
|  |  | (*c*) Explain how missing data were addressed | 6 |
|  |  | (*d*) If applicable, explain how loss to follow-up was addressed | 5 |
|  |  | (*e*) Describe any sensitivity analyses | 6-7 |
| Results | | |  |
| Participants | 13* | (a) Report numbers of individuals at each stage of study—eg numbers potentially eligible, examined for eligibility, confirmed eligible, included in the study, completing follow-up, and analysed | 8 |
|  |  | (b) Give reasons for non-participation at each stage | NA |
|  |  | (c) Consider use of a flow diagram | NA |
| Descriptive data | 14* | (a) Give characteristics of study participants (eg demographic, clinical, social) and information on exposures and potential confounders | 8 |
|  |  | (b) Indicate number of participants with missing data for each variable of interest | 9 |
|  |  | (c) Summarise follow-up time (eg, average and total amount) | NA |
| Outcome data | 15* | Report numbers of outcome events or summary measures over time | 8 |
| Main results | 16 | (*a*) Give unadjusted estimates and, if applicable, confounder-adjusted estimates and their precision (eg, 95% confidence interval). Make clear which confounders were adjusted for and why they were included | 8-11 |
|  |  | (*b*) Report category boundaries when continuous variables were categorized | 8-11 |
|  |  | (*c*) If relevant, consider translating estimates of relative risk into absolute risk for a meaningful time period | NA |
| Other analyses | 17 | Report other analyses done—eg analyses of subgroups and interactions, and sensitivity analyses | ?NA |
| Discussion | | |  |
| Key results | 18 | Summarise key results with reference to study objectives | 12 |
| Limitations | 19 | Discuss limitations of the study, taking into account sources of potential bias or imprecision. Discuss both direction and magnitude of any potential bias | 13 |
| Interpretation | 20 | Give a cautious overall interpretation of results considering objectives, limitations, multiplicity of analyses, results from similar studies, and other relevant evidence | 12-13 |
| Generalisability | 21 | Discuss the generalisability (external validity) of the study results | 12-13 |
| Other information | | |  |
| Funding | 22 | Give the source of funding and the role of the funders for the present study and, if applicable, for the original study on which the present article is based | 7 |

*Give information separately for exposed and unexposed groups.

**Note:** An Explanation and Elaboration article discusses each checklist item and gives methodological background and published examples of transparent reporting. The STROBE checklist is best used in conjunction with this article (freely available on the Web sites of PLoS Medicine at http://www.plosmedicine.org/, Annals of Internal Medicine at http://www.annals.org/, and Epidemiology at http://www.epidem.com/). Information on the STROBE Initiative is available at http://www.strobe-statement.org.

**References**

1. Kimura Y, Takada T, Kawarada Y, Nimura Y, Hirata K, Sekimoto M, Yoshida M, Mayumi T, *et al* (2007). *Definitions, Pathophysiology, and Epidemiology of Acute Cholangitis and Cholecystitis: Tokyo Guidelines*. *J Hepatobiliary Pancreat Surg* 14: 15–26. https://doi.org/10.1007/s00534-006-1152-y.

2. Pisano M, Allievi N, Gurusamy K, Borzellino G, Cimbanassi S, Boerna D, Coccolini F, Tufo A, *et al* (2020). *2020 World Society of Emergency Surgery Updated Guidelines for the Diagnosis and Treatment of Acute Calculus Cholecystitis*. *World J Emerg Surg* 15: 61. https://doi.org/10.1186/s13017-020-00336-x.

3. Okamoto K, Suzuki K, Takada T, Strasberg SM, Asbun HJ, Endo I, Iwashita Y, Hibi T, *et al* (2018). *Tokyo Guidelines 2018: Flowchart for the Management of Acute Cholecystitis*. *J Hepatobiliary Pancreat Sci* 25: 55–72. https://doi.org/10.1002/jhbp.516.

4. Cameron IC (2004). *Management of Acute Cholecystitis in UK Hospitals: Time for a Change*. *Postgrad Med J* 80: 292–4. https://doi.org/10.1136/pgmj.2002.004085.

5. Mytton J, Daliya P, Singh P, Parsons SL, Lobo DN, Lilford R, Vohra RS (2019). *Outcomes Following an Index Emergency Admission with Cholecystitis*. *Ann Surg* 274. https://doi.org/10.1097/SLA.0000000000003599.

6. Loozen CS, Oor JE, van Ramshorst B, van Santvoort HC, Boerma D (2017). *Conservative Treatment of Acute Cholecystitis: A Systematic Review and Pooled Analysis*. *Surg Endosc* 31: 504–15. https://doi.org/10.1007/s00464-016-5011-x.

7. Brazzelli M, Cruickshank M, Kilonzo M, Ahmed I, Stewart F, McNamee P, Elders A, Fraser C, *et al* (2014). *Clinical Effectiveness and Cost-Effectiveness of Cholecystectomy Compared with Observation/Conservative Management for Preventing Recurrent Symptoms and Complications in Adults Presenting with Uncomplicated Symptomatic Gallstones or Cholecystitis: A Syste*. *Health Technol Assess (Rockv)* 18: 1–102. https://doi.org/10.3310/hta18550.

8. Abdel-Salam WN, Bekheit M, Katri K, Ezzat T, El Kayal ES (2013). *Efficacy of Intragastric Balloon in Obese Egyptian Patients and the Value of Extended Liquid Diet Period in Mounting the Weight Loss.* *J Laparoendosc Adv Surg Tech A* 23: 220–4.

9. Scottish Government (2018). *Scottish Government Urban Rural Classification 2016.* Available at: https://www.gov.scot/publications/scottish-government-urban-rural-classification-2016/pages/2/.

10. Ramkumar N, Colla CH, Wang Q, O’Malley AJ, Wong SL, Brooks GA (2022). *Association of Rurality, Race and Ethnicity, and Socioeconomic Status With the Surgical Management of Colon Cancer and Postoperative Outcomes Among Medicare Beneficiaries*. *JAMA Netw Open* 5: e2229247–e2229247. https://doi.org/10.1001/jamanetworkopen.2022.29247.

11. Government S (2016). *Introducing The Scottish Index of Multiple Deprivation 2016. A National Statistics Publication for Scotland.*

12. Wohlgemut JM, Ramsay G, Griffin RL, Jansen JO (2020). *Impact of Deprivation and Comorbidity on Outcomes in Emergency General Surgery: An Epidemiological Study*. *Trauma Surg Acute Care Open* 5: e000500. https://doi.org/10.1136/tsaco-2020-000500.

13. Reddy SR, Soonawalla Z, Silva MA (2018). *Acalculous Cholecystitis : Is an Elective Interval Cholecystectomy Necessary ?*. 171–6. https://doi.org/10.1159/000477780.

14. Aroori S, Mangan C, Reza L, Gafoor N (2019). *Percutaneous Cholecystostomy for Severe Acute Cholecystitis: A Useful Procedure in High-Risk Patients for Surgery*. *Scand J Surg* 108: 124–9. https://doi.org/10.1177/1457496918798209.

15. Li F, Zaslavsky A, Landrum M (2007). *Propensity Score Analysis with Hierarchical Data*. *Proc Am …*: 2474–81.

16. von Elm E, Altman DG, Egger M, Pocock SJ, Gøtzsche PC, Vandenbroucke JP, STROBE Initiative (2007). *The Strengthening the Reporting of Observational Studies in Epidemiology (STROBE) Statement: Guidelines for Reporting Observational Studies.* *Lancet (London, England)* 370: 1453–7. https://doi.org/10.1016/S0140-6736(07)61602-X.

17. Wohlgemut JM, Ramsay G, Bekheit M, Scott NW, Watson AJM, Jansen JO (2021). *Emergency General Surgery: Impact of Hospital and Surgeon Admission Case Volume on Mortality*. *J Trauma Acute Care Surg* 90: 996–1002. https://doi.org/10.1097/TA.0000000000003128.

18. Ramsay G, Wohlgemut JM, Jansen JO (2018). *Emergency General Surgery in the United Kingdom: A Lot of General, Not Many Emergencies, and Not Much Surgery*. *J Trauma Acute Care Surg* 85: 500–6. https://doi.org/10.1097/TA.0000000000002010.

19. Filiberto AC, Efron PA, Frantz A, Bihorac A, Upchurch GR, Loftus TJ (2022). *Personalized Decision-Making for Acute Cholecystitis: Understanding Surgeon Judgment*. *Front Digit Heal* 4: 1–10. https://doi.org/10.3389/fdgth.2022.845453.

20. Bedirli A, Sakrak O, Sözüer EM, Kerek M, Güler I. *Factors Effecting the Complications in the Natural History of Acute Cholecystitis.* *Hepatogastroenterology* 48: 1275–8.

21. FELÍCIO SJO, MATOS EP, CERQUEIRA AM, FARIAS KWSF de, SILVA R de A, TORRES M de O (2017). *MORTALITY OF URGENCY VERSUS ELECTIVE VIDEOLAPAROSCOPIC CHOLECYSTECTOMY FOR ACUTE CHOLECYSTITIS*. *ABCD Arq Bras Cir Dig (São Paulo)* 30: 47–50. https://doi.org/10.1590/0102-6720201700010013.

22. Gallaher JR, Charles A (2022). *Acute Cholecystitis*. *JAMA* 327: 965. https://doi.org/10.1001/jama.2022.2350.

23. Escartín A, González M, Cuello E, Pinillos A, Muriel P, Merichal M, Palacios V, Escoll J, *et al* (2019). *Acute Cholecystitis in Very Elderly Patients: Disease Management, Outcomes, and Risk Factors for Complications*. *Surg Res Pract* 2019: 9709242. https://doi.org/10.1155/2019/9709242.

24. Loozen CS, Van Santvoort HC, Van Duijvendijk P, Besselink MG, Gouma DJ, Nieuwenhuijzen GA, Kelder JC, Donkervoort SC, *et al* (2018). *Laparoscopic Cholecystectomy versus Percutaneous Catheter Drainage for Acute Cholecystitis in High Risk Patients (CHOCOLATE): Multicentre Randomised Clinical Trial*. *BMJ* 363. https://doi.org/10.1136/bmj.k3965.

25. Yokoe M, Takada T, Hwang T-L, Endo I, Akazawa K, Miura F, Mayumi T, Mori R, *et al* (2017). *Validation of TG13 Severity Grading in Acute Cholecystitis: Japan-Taiwan Collaborative Study for Acute Cholecystitis*. *J Hepatobiliary Pancreat Sci* 24: 338–45. https://doi.org/10.1002/jhbp.457.

26. Sandblom G, Videhult P, Crona Guterstam Y, Svenner A, Sadr-Azodi O (2015). *Mortality after a Cholecystectomy: A Population-Based Study*. *Hpb* 17: 239–43. https://doi.org/10.1111/hpb.12356.
